# Intralysosomal pathogens differentially influence the proteolytic potential of their niche

**DOI:** 10.1128/iai.00270-25

**Published:** 2025-12-30

**Authors:** Lauren E. Bird, Bangyan Xu, David R. Thomas, Erin N. S. McGowan, Patrice Newton, Nichollas E. Scott, Malcolm J. McConville, Laura E. Edgington-Mitchell, Hayley J. Newton

**Affiliations:** 1 Department of Microbiology and Immunology at the Peter Doherty Institute for Infection and Immunity, The University of Melbourne 2281, Melbourne, Victoria, Australia; 2 Department of Microbiology, Infection Program, Monash Biomedicine Discovery Institute 2541, Melbourne, Victoria, Australia; 3 Department of Biochemistry and Pharmacology, Bio21 Molecular Science and Biotechnology Institute, The University of Melbourne 2281, Melbourne, Victoria, Australia; University of Pennsylvania Perelman School of Medicine, Philadelphia, Pennsylvania, USA

**Keywords:** *Coxiella burnetii*, *Leishmania mexicana*, cathepsin B, cathepsin D, co-infection

## Abstract

Avoiding lysosomal degradation is vital to the success of intracellular pathogens. The Gram-negative bacterium *Coxiella burnetii* and protozoan parasites of the *Leishmania* genus are unique in being able to replicate within the mature phagolysosomal compartment of host cells, though the exact mechanisms utilized to withstand this hostile environment are not clearly defined. We recently reported that *C. burnetii* removes the lysosomal protease cathepsin B during infection of mammalian cells. Here, we aimed to determine if this virulence strategy was also employed by the intralysosomal pathogen, *Leishmania mexicana*. In contrast to *C. burnetii*, decreases in the activity of specific cathepsins were not detected in *L. mexicana*-infected host cells as determined using immunoblotting and protease activity-based probes. Co-infection of THP-1 macrophage-like cells with both pathogens resulted in a proteolytic and secretory phenotype consistent with *C. burnetii* infection, suggesting that *C. burnetii-*induced remodeling of the lysosome is not influenced by *L. mexicana*. The host cell proteome and secretome of *L. mexicana*-infected cells were defined using mass spectrometry. This confirmed that, unlike *C. burnetii*, *L. mexicana* does not induce increased abundance of lysosomal proteins either intracellularly or in the extracellular milieu. Collectively, this study reveals that although *C. burnetii* and *L. mexicana* reside in a phagolysosomal intracellular niche, they employ divergent mechanisms to survive within this hostile compartment.

## INTRODUCTION

Evading lysosomal degradation is of critical importance to successful intracellular pathogens. The lysosome, classically considered the waste disposal and recycling center of the cell, is a hostile environment consisting of many hydrolytic enzymes which serve to break down macromolecules to maintain cellular homeostasis. Most intracellular pathogens have evolved strategies to avoid delivery to lysosomes, either by halting maturation of the pathogen-containing phagosome (e.g., *Legionella pneumophila, Mycobacterium tuberculosis*) (1), escaping into the cytosol for replication (*Rickettsia*, *Listeria, Shigella* spp.) (2), or generating a unique, non-endocytic compartment (e.g., *Chlamydia* spp.) (3). The only known human pathogens that replicate inside a lysosome-derived niche are the protozoa *Leishmania* spp. and the Gram-negative bacterium *Coxiella burnetii*.

*Leishmania* spp. are protozoan parasites that cause the devastating and potentially fatal spectrum of diseases collectively termed “leishmaniases” (reviewed in reference 4). Symptoms of leishmaniasis can range from self-healing cutaneous lesions to chronic, multisystemic disease depending on the parasite species responsible for infection. An estimated 700,000–1 million new cases occur annually, with the World Health Organization listing leishmaniases as a class of major neglected tropical diseases (5).

*Leishmania* parasites exhibit a digenetic lifecycle. The extracellular, flagellated promastigote replicates in the midgut of the sandfly and is transmitted to a vertebrate host during a blood meal. Promastigotes are internalized by phagocytic cells and subsequently differentiate into aflagellated, non-motile amastigotes, which replicate inside an intracellular parasitophorous vacuole (PV) with phagolysosomal features (6). Macrophages and monocytes are the primary cell type infected by *Leishmania,* though parasites can also enter and persist in neutrophils and dendritic cells, as well as non-phagocytic cells (7, 8). Following phagocytosis, parasites are delivered to a mature phagolysosome which continues to fuse with other vesicles of the endolysosomal system. Some *Leishmania* species (e.g., *L. amazonensis, L. mexicana*) generate large communal phagolysosomes containing hundreds of parasites, while other species (e.g., *L. major*) reside within individual, tight-fitting phagolysosomes (9). During maturation, *Leishmania* PVs sequentially acquire the endosomal markers Rab5 and Rab7, as well as lysosomal membrane proteins (LAMP-1, LAMP-2) and hydrolases (cathepsins) (10, 11). The promastigote stages of some species of *Leishmania* may transiently delay the maturation of the PV, although this is not evident with *L. mexicana* promastigotes or the amastigote stages of all species (reviewed in reference 12). How *Leishmania* amastigotes avoid hydrolytic degradation in a lysosome-derived compartment remains poorly understood.

The only other known pathogen that requires a lysosomal environment for replication is the Gram-negative bacterium *C. burnetii. C. burnetii* is the causative agent of Q fever, which has a spectrum of clinical presentations ranging from asymptomatic seroconversion to chronic, life-threatening illness (13). Human infection occurs through inhalation of contaminated aerosols, and the bacterium establishes its replicative niche inside alveolar macrophages of infected individuals. The mature pathogen-containing compartment is termed the *Coxiella-*containing vacuole (CCV) that displays both endolysosomal (Rab5, Rab7, LAMP-1 and 2) and autophagosomal (LC3, p62) markers (14). From within this replicative niche, *C. burnetii* employs a type IV-B secretion system (T4SS) to translocate approximately 130 bacterial proteins into the host cytosol to manipulate various host processes which contribute to infection (15).

The *C. burnetii* CCV demonstrates remarkable fusogenicity and has been previously shown to take up inert latex beads or other pathogens including *Trypanosoma cruzi* (16) and *Mycobacterium avium* (17). There is also existing evidence that *C. burnetii* and *L. amazonensis* can cohabit a single vacuole within a mammalian cell. It has been demonstrated that *L. amazonensis* can traffic to and replicate within pre-existing *C. burnetii* vacuoles in Chinese Hamster Ovary cells (18), and that Vero cells infected with *L. amazonensis* can be super-infected with Dot/Icm-deficient *C. burnetii* to promote intracellular proliferation of this normally non-replicative strain (19). This suggests that the vacuoles housing *C. burnetii* and *L. amazonensis* are at least superficially similar. Whether this is true of other *Leishmania* species is unclear.

We recently reported that *C. burnetii* removes the lysosomal protease cathepsin B from infected cells, leading to improved bacterial replication and intracellular success (20). In this study, we have investigated whether *L. mexicana* similarly manipulates the levels of specific lysosomal proteases. Using a panel of activity-based probes (ABPs) and immunoblotting, it was found that *C. burnetii* and *L. mexicana* differentially modulate the proteolytic capacity of their replicative niche. Additionally, *L. mexicana* infection did not induce the extracellular secretion of lysosomal content, as previously observed for *C. burnetii* (20). Collectively, this indicates that although these pathogens reside inside a niche with superficially similar characteristics, they modulate the cohort of lysosomal proteases in distinct ways to promote their own intracellular survival.

## RESULTS

### Cathepsin abundance and activity are differentially modulated by *C. burnetii* and *L. mexicana*

**Fig 1:**
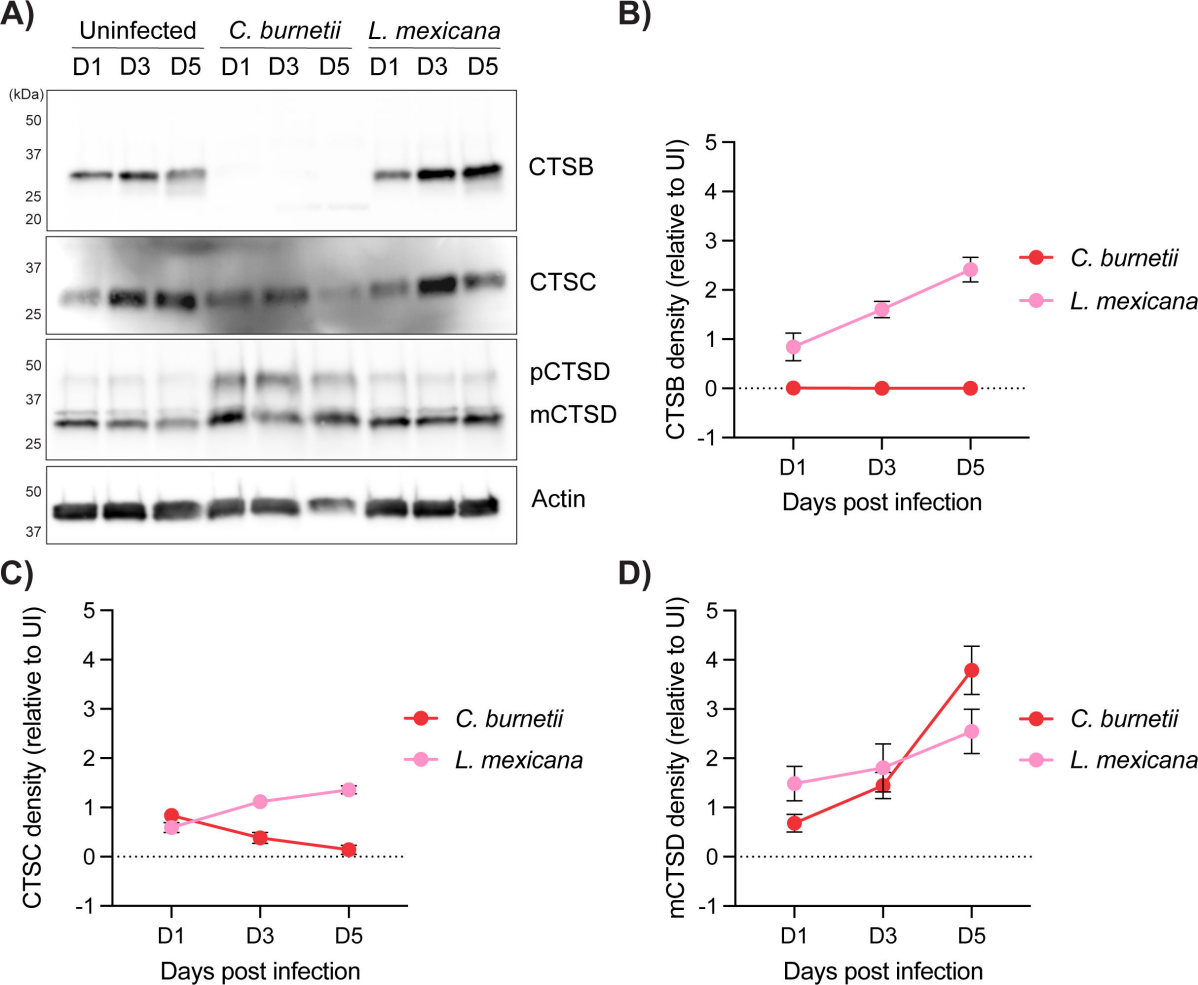
Cathepsin abundance is differentially modified by *C. burnetii* and *L. mexicana*. (**A**) THP-1 cells were left uninfected or infected for 5 days with *C. burnetii* or *L. mexicana* at an MOI of 10. At days 1, 3, or 5 post-infection, cells were lysed and subjected to SDS-PAGE and immunoblotting using antibodies against cathepsin B (CTSB), cathepsin C (CTSC), cathepsin D (CTSD), or actin as a loading control. pCTSD, pro-cathepsin D; mCTSD, mature cathepsin D. Blot is representative of three independent replicates. (**B**) Quantification of cathepsin B density as presented in panel **A**. Values are expressed relative to uninfected cells at the same time point and standardized against respective actin expression. The graph depicts the average of three biological replicates. Error bars represent standard deviation. (**C**) Quantification of cathepsin C density as presented in panel **A**. Values are expressed relative to uninfected cells at the same time point and standardized against respective actin expression. The graph depicts the average of three biological replicates. Error bars represent standard deviation. (**D**) Quantification of mCTSD density as presented in panel **A**. Values are expressed relative to uninfected cells at the same time point and standardized against respective actin expression. The graph depicts the average of three biological replicates. Error bars represent standard deviation.

*C. burnetii* elicits removal of the lysosomal protease cathepsin B from mammalian cells to promote infection (20). To explore whether this is a shared strategy of intralysosomal pathogens, the abundance of specific cathepsins was measured during infection with *L. mexicana. L. mexicana* was chosen as a representative leishmanial species because of the similar fusogenicity of the *L. mexicana* PV to the *C. burnetii* CCV (multiple parasites within a single communal vacuole) and the ability to work with this pathogen under Biosafety Level 2 conditions. Differentiated THP-1 cells were infected with either *C. burnetii* or *L. mexicana* at a multiplicity of infection (MOI) of 10, and the abundance of specific cathepsins was assessed over 5 days of infection. Consistent with previous observations, cathepsin B was undetectable in *C. burnetii-*infected cells (Fig. 1A and B). In contrast, THP-1 cells infected with *L. mexicana* exhibited an increase in cathepsin B abundance over the infection time course (Fig. 1A and B). To determine if this was the case for other cathepsins, we immunoblotted for cathepsins C and D. Consistent with previous findings (20), *C. burnetii* infection led to a reduction in cathepsin C (Fig. 1A and C), while *L. mexicana* infection resulted in somewhat increased cathepsin C abundance (Fig. 1A and C). The pro-form of cathepsin D (pCTSD) accumulated in *C. burnetii-*infected cells, but not uninfected or *L. mexicana*-infected cells (Fig. 1A), while mature cathepsin D (mCTSD) gradually accumulated over time in both infection conditions (Fig. 1D).

**Fig 2:**
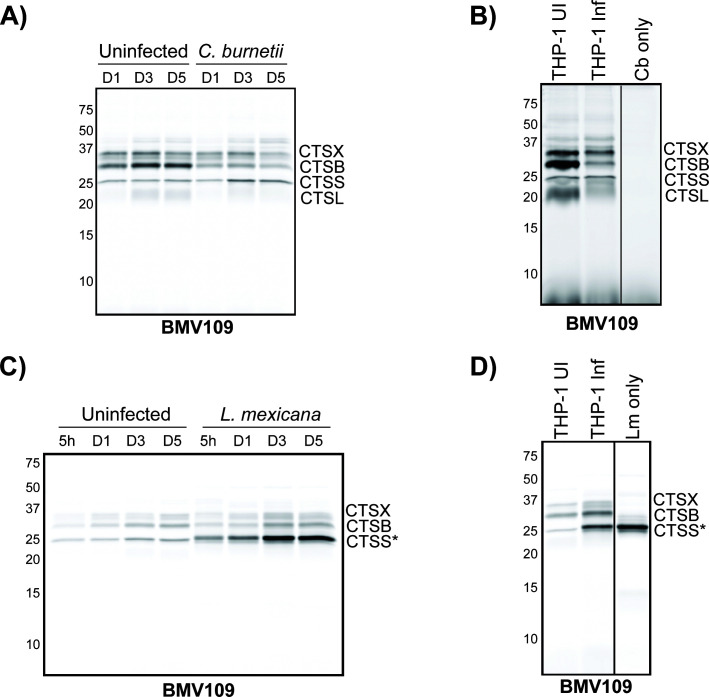
ABP profiling of cathepsin proteases during *C. burnetii* or *L. mexicana* infection of THP-1 cells. (**A**) THP-1 cells were uninfected or infected with *C. burnetii* for 5 days at an MOI of 10. At days 1, 3, or 5 post-infection, cells were lysed in a citrate buffer and active cysteine proteases labeled with 1 μM BMV109 before being resolved by SDS-PAGE. The image depicts in-gel fluorescence of BMV109-labeled cysteine proteases from uninfected or *C. burnetii*-infected THP-1 cells at days 1, 3, or 5 post-infection. (**B**) In-gel fluorescence of BMV109-labeled cysteine proteases from uninfected THP-1 cells (“THP-1 UI”), 3 day *C*. *burnetii-*infected cells (“THP-1 Inf”), or lysed *C. burnetii* (“Cb only”). (**C**) Same as in panel **A**, but cells were infected with *L. mexicana* amastigotes at an MOI of 10. Lysates were harvested at 5 h, 1, 3, or 5 days post-infection. “*” denotes parasitic protease at the same molecular weight as CTSS. (**D**) In-gel fluorescence of BMV109-labeled cysteine proteases from uninfected THP-1 cells (“THP-1 UI”), 3 day *L*. *mexicana*-infected cells (“THP-1 Inf”), or lysed *L. mexicana* amastigotes (“Lm only”). “*” denotes a parasitic protease at the same molecular weight as CTSS. CTSX, cathepsin X; CTSB, cathepsin B; CTSS, cathepsin S; CTSL, cathepsin L.

Given that protein abundance does not necessarily correlate with enzymatic activity, these investigations were extended to examine cathepsin activity in infected cells by leveraging the ABP BMV109 (21, 22). ABPs are small molecules that covalently bind to the active site of proteases and allow detection of enzyme activity through in-gel fluorescence (23). BMV109 is a pan-reactive probe for cysteine cathepsin activity, allowing simultaneous detection of cathepsin B, S, X, and L activity in a single sample (21, 22). Incubation of *C. burnetii*-infected cell lysates with BMV109 again confirmed the complete loss of cathepsin B activity, consistent with its reduced abundance from cells (Fig. 2A). Other cysteine cathepsins appeared broadly unchanged during *C. burnetii* infection. Furthermore, no labeling was observed when this probe was incubated with lysed *C. burnetii* alone (Fig. 2B), confirming that BMV109 is specifically labeling mammalian and not *C. burnetii*-derived proteases. Interestingly, when BMV109 was applied to lysates from *L. mexicana-*infected cells, increased activity of a protease at a similar size to cathepsin S was observed (Fig. 2C, “CTSS*”). This activity is likely to be associated with the well-characterized cysteine proteases in *L. mexicana* amastigotes (24), which was confirmed by incubating lysed *L. mexicana* amastigotes (no host cells) with the probe (Fig. 2D). The other cysteine cathepsins identified with BMV109 labeling showed unchanged activity during *L. mexicana* infection (Fig. 2C). Collectively, the data presented in Fig. 1 and 2 suggest that *C. burnetii* and *L. mexicana* differentially regulate the levels of host lysosomal proteases, despite residing within intracellular vacuoles with similar characteristics.

### Co-infection with *C. burnetii* and *L. mexicana* does not alter replication dynamics or protease abundance

**Fig 3:**
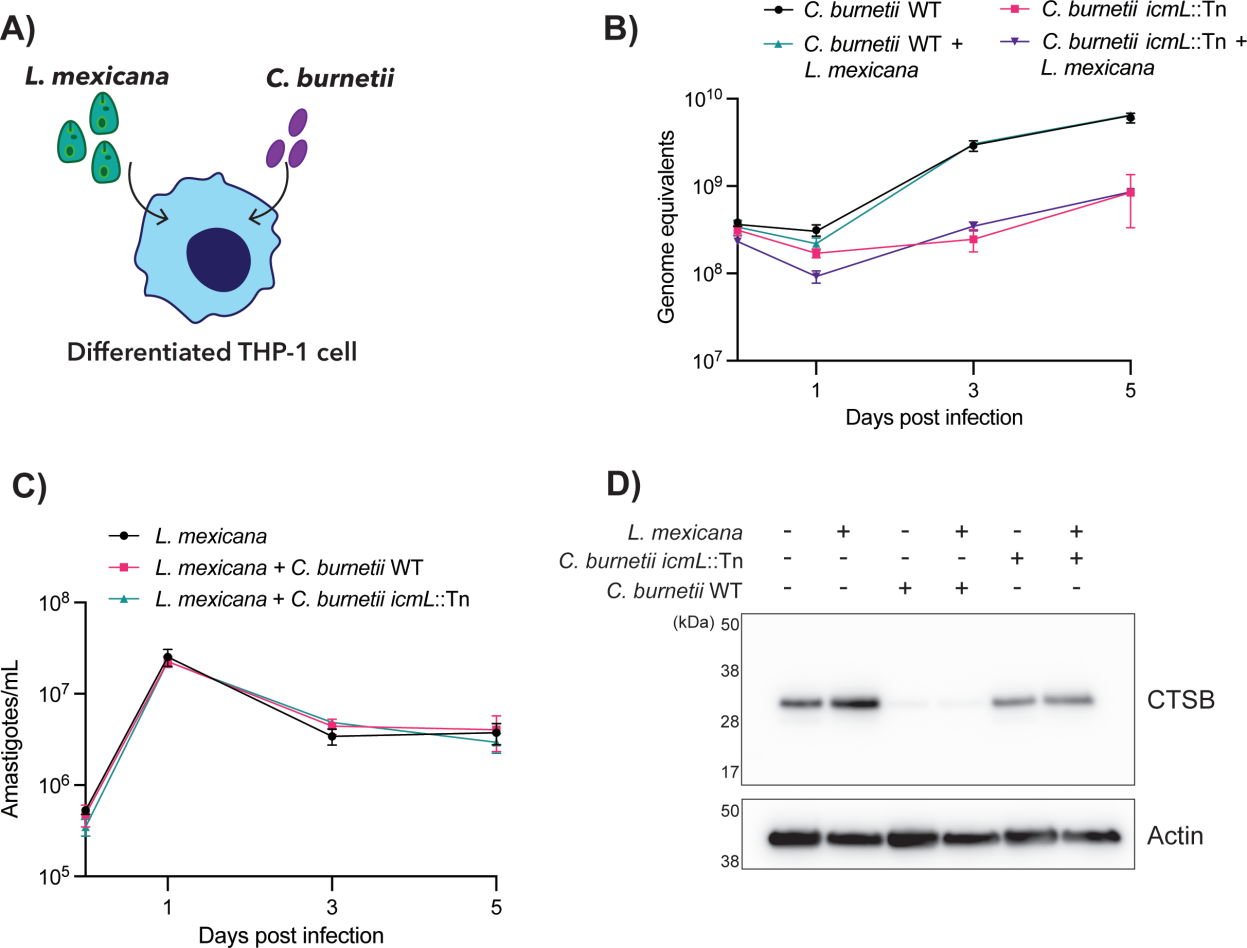
Co-infection with *C. burnetii* and *L. mexicana* does not impact replication dynamics or protease abundance. (**A**) Schematic of experimental design. (**B**) qPCR analysis of *C. burnetii* intracellular replication in THP-1 cells infected with the indicated strains ± *L. mexicana*. Genome equivalents were calculated based on a standard curve. Individual data points represent the mean of three biological replicates. Error bars denote standard deviation. (**C**) qPCR analysis of *L. mexicana* intracellular replication in THP-1 cells infected ± *C. burnetii* (wild-type or *icmL*::Tn strains). Individual data points represent the mean of three biological replicates. Error bars denote standard deviation. (**D**) THP-1 cells were uninfected or infected with the indicated pathogen(s) at an MOI of 10. At 3 days post-infection, lysates were harvested and subjected to SDS-PAGE and immunoblotting. CTSB, cathepsin B.

We next aimed to establish a co-infection model whereby cells were simultaneously infected with *C. burnetii* (either wild-type or a T4SS-deficient mutant, *icmL*::Tn) and *L. mexicana* (Fig. 3A). In our previous work, *C. burnetii* replication was negatively impacted by overexpression of cathepsin B (20). Since cathepsin B is retained during *L. mexicana* infection (Fig. 1), we speculated that co-infection might similarly affect *C. burnetii* replication.

To assess replication in co-infected cells, real-time quantitative PCR was performed using primers specific to the *C. burnetii* constitutively expressed *ompA* gene, as described previously (25). Using this approach, no change to *C. burnetii* replication dynamics was observed, regardless of *L. mexicana* co-infection (Fig. 3B). The same approach was used to quantify intracellular replication of *L. mexicana,* using primers targeting the kinetoplast DNA minicircle (26). A sharp increase in parasite load was observed between day 0 and day 1 post-infection, followed by a slight drop and plateau for the remainder of the time course (Fig. 3C). Co-infection with either strain of *C. burnetii* did not significantly alter these dynamics.

Samples from day 3 of these infections were immunoblotted for cathepsin B. As before, a dramatic decrease in cathepsin B abundance was observed when cells were infected with *C. burnetii*, and this was not restored when *L. mexicana* was present. Infection with a *C. burnetii* T4SS mutant (*icmL*::Tn) led to cathepsin B retention in both singly infected and co-infected conditions. Taken together, these data indicate that *C. burnetii* maintains the ability to remove cathepsin B and replicate to equivalent numbers during co-infection as when infected alone.

### A *C. burnetii* T4SS mutant undergoes limited replication in *L. mexicana-*infected cells

**Fig 4:**
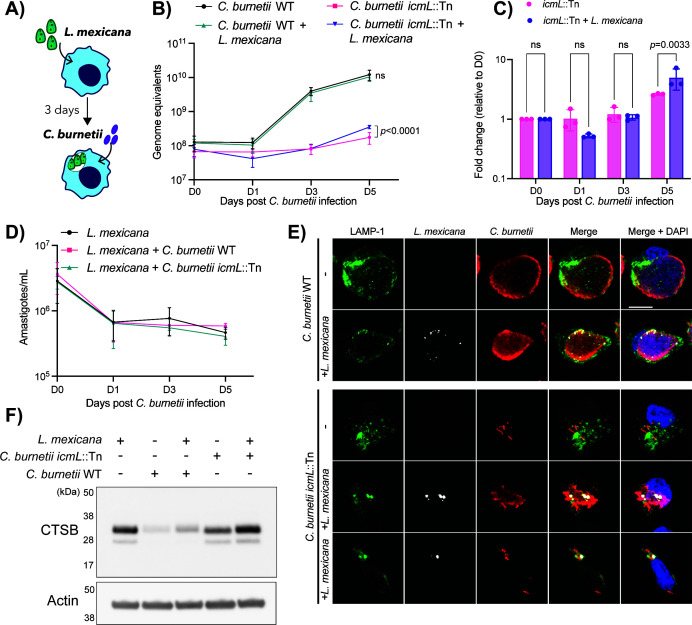
T4SS-deficient *C. burnetii* exhibit increased replication in *L. mexicana-*infected cells. (**A**) Schematic of experimental design. (**B**) qPCR analysis of *C. burnetii* intracellular replication in THP-1 cells infected with the indicated strains ± *L. mexicana*. Genome equivalents were calculated based on a standard curve. Individual data points represent the mean of three biological replicates. Error bars denote standard deviation. (**C**) Data from panel **B** but presented as fold change in *C. burnetii* replication relative to genome equivalents present at day 0. (**D**) qPCR analysis of *L. mexicana* intracellular replication in THP-1 cells infected ± *C. burnetii* (wild-type or *icmL*::Tn strains). Individual data points represent the mean of three biological replicates. Error bars denote standard deviation. (**E**) Representative confocal images of THP-1 cells infected with *L. mexicana* or remaining uninfected for 3 days before infection with *C. burnetii* strains for 5 days. LAMP-1 (green) denotes the pathogen-containing vacuoles with *C. burnetii* shown in red and *L. mexicana* in white. *C. burnetii icmL*::Tn led to two distinct phenotypes, with some co-inhabited vacuoles supporting replication of this strain. Scale bar = 10 μM. (**F**) THP-1 cells were infected with *L. mexicana* for 3 days and then super-infected with *C. burnetii* for a further 3 days. At this time, lysates were harvested and subjected to SDS-PAGE and immunoblotting. CTSB, cathepsin B.

No change to the replication dynamics of *C. burnetii* was observed when cells were simultaneously infected with both microbes. However, we wondered if this might be altered by sequential infection. It has previously been reported that *C. burnetii* can traffic to pre-existing vacuoles established by *L. amazonensis* and that this restored the replication defect observed in a T4SS-deficient *C. burnetii* strain (19). However, the extent to which *C. burnetii* T4SS mutants can replicate inside a *Leishmania* vacuole has never been quantified. Given this, we established a super-infection model, where THP-1 cells were infected for 3 days with *L. mexicana* before being super-infected with *C. burnetii* (wild-type or *icmL*::Tn) (Fig. 4A). Intracellular replication of both pathogens was monitored as outlined above. The presence of a pre-existing *L. mexicana* vacuole did not alter the replication kinetics of wild-type *C. burnetii* but did lead to a significant increase in replication of *icmL*::Tn at 5 days post *C. burnetii* infection (Fig. 4B). When quantified as fold change in replication relative to bacterial load at day 0, we observed a significant increase in *C. burnetii icmL*::Tn replication at day 5 post-infection when *L. mexicana* was also present (mean fold change 5.021 ± 1.947) compared to when cells were infected with *icmL*::Tn alone (mean fold change 2.620 ± 0.100; two-way ANOVA with Sidak’s correction, *P* = 0.0033; Fig. 4C). Quantification of *L. mexicana* across this time course revealed a decrease in parasite load at day 1 post *C. burnetii* infection (day 4 post *L. mexicana* infection), followed by a plateau in *L. mexicana* numbers (Fig. 4D). Confocal microscopy was employed to further characterize the dynamics of these super-infections. THP-1 cells were infected with *L. mexicana* expressing TurboRFP and three days later were super-infected with *C. burnetii* strains before being fixed and stained for LAMP-1 and *C. burnetii* 5 days post-infection. Representative images are shown (Fig. 4E). Typical large CCVs were observed for *C. burnetii* WT in the presence or absence of *L. mexicana*. The *C. burnetii icml*::Tn strain was observed as individual, intracellular bacteria in the majority of super-infected cells; however, some cells were observed harboring multiple Dot/Icm-deficient *C. burnetii* co-inhabiting a vacuole with *L. mexicana*.

Examination of cathepsin B abundance was performed at 3 days following *C. burnetii* infection (5 days following *L. mexicana* infection). Similar to what was observed during co-infection, cathepsin B levels were decreased when super-infected THP-1 cells were infected with wild-type *C. burnetii*, but not with *icmL*::Tn (Fig. 4F). The presence of a pre-existing *L. mexicana* infection did not noticeably alter this. Altogether, these data indicate that a non-replicative T4SS mutant exhibits a small but significant increase in replication in a pre-established *L. mexicana* vacuole, though this is not due to changes in cathepsin abundance.

### *L. mexicana* does not induce secretion of lysosomal content

**Fig 5:**
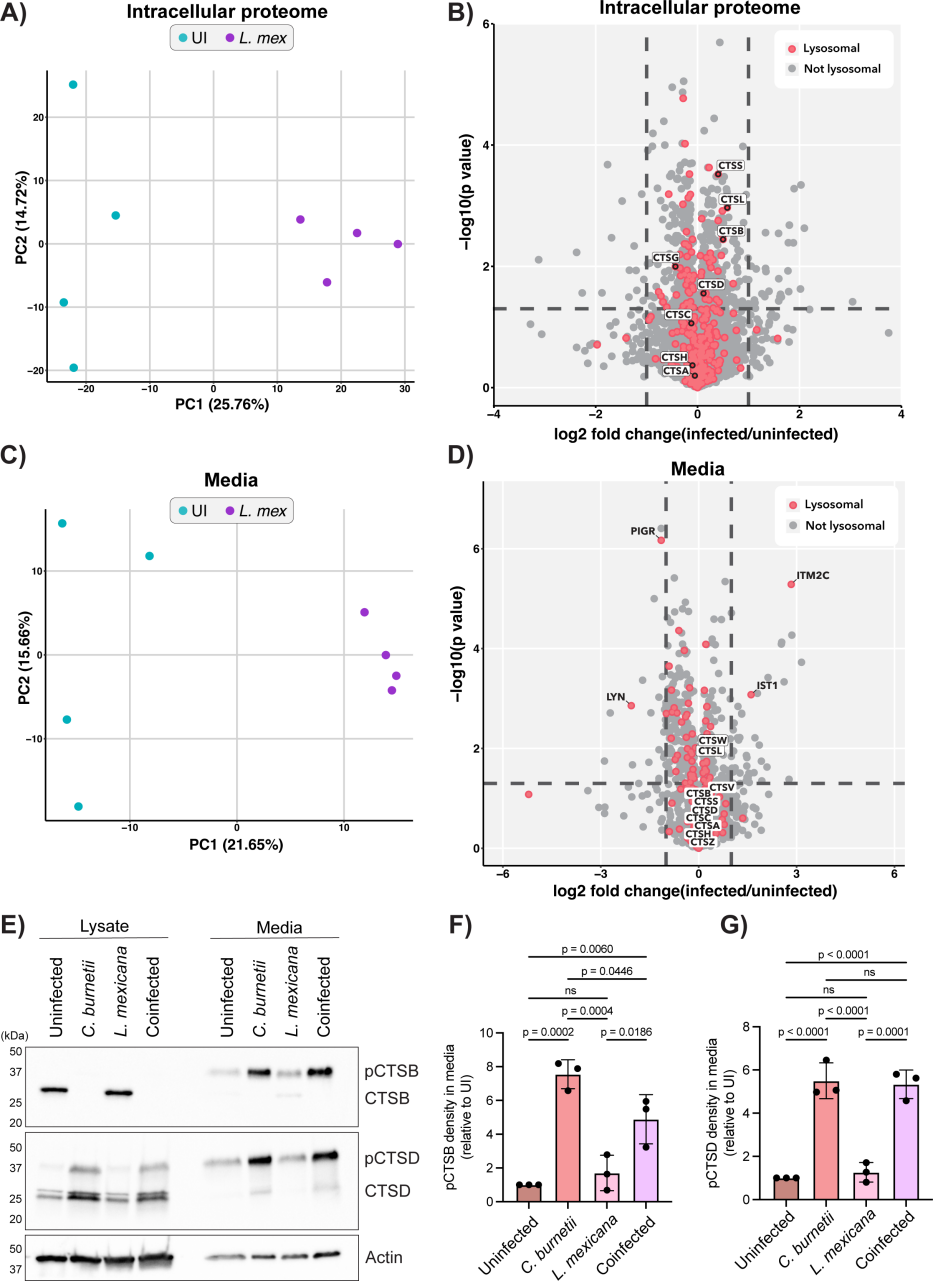
*L. mexicana* does not induce extracellular secretion of lysosomal proteins. (**A–D**) THP-1 cells were left uninfected (*n* = 4) or infected with *L. mexicana* (*n* = 4) at an MOI of 10 for 2 days before being washed and changed to serum-free culture media for a further 18 h. At this time, cell lysates and conditioned media were harvested and subjected to label-free quantitative mass spectrometry. (**A**) PCA plot of proteomics data from whole cell lysates (“intracellular proteome”). Uninfected samples are shown in teal; *L. mexicana*-infected samples are shown in purple (*n* = 4/group). (**B**) Volcano plot of proteins enriched in the intracellular proteome of *L. mexicana*-infected THP-1 cells as determined by proteomics analysis. *X*-axis represents log_2_ (fold change) relative to uninfected cells; *Y*-axis shows statistical significance, −log_10_ (*P*-value, as determined by Student’s *t*-test). Proteins with designated lysosomal localization are colored in pink; cathepsins and significantly altered lysosomal proteins are labeled with gene names. (**C**) Same as panel **A** but depicting PCA plot of samples from conditioned media of *L. mexicana*-infected THP-1 cells. (**D**) Same as panel **B** but volcano plot of proteins enriched in the conditioned media from *L. mexicana*-infected THP-1 cells. (**E**) Immunoblot depicting cathepsin expression in the lysates and media of cells infected with *C. burnetii, L. mexicana,* or co-infected with both microbes simultaneously. Blot is representative of three independent experiments, quantified in panels **F and G**. (**F**) Quantification of pro-cathepsin B (pCTSB) density in media as presented in panel **E**. Values are expressed relative to uninfected cells and standardized against respective actin expression. The graph depicts three biological replicates. Error bars denote SD. (**G**) Same as panel **F** but depicting pCTSD density in media. *P*-values were calculated using one-way ANOVA with Tukey’s correction. Mass spectrometry source data are available via ProteomeXchange with accession number PXD063745.

*C. burnetii* infection causes a T4SS-independent secretion of lysosomal content into the extracellular milieu (20). To determine if a similar phenotype was observed upon *L. mexicana* infection, proteomics analysis was performed on both the lysate and conditioned media collected from THP-1 cells after 3 days of *L. mexicana* infection (Data S1). Using data-dependent acquisition label-free proteomics, 4,266 proteins were identified and quantified with a false discovery rate (FDR) of 1% in the whole cell lysate samples. Of these, 4,098 were host-derived and 168 were derived from *L. mexicana*. Principal component analysis (PCA) showed that biological replicates from each condition clustered together (Fig. 5A). Only a small number of changes to the host proteome were observed during infection, with 64 proteins significantly increased in abundance and 23 proteins showing decreased abundance, as determined by a Student’s *t*-test with *P* < 0.05 and log_2_ fold change > 1 (Fig. 5B). *C. burnetii* infection leads to increased abundance of lysosomal proteins, likely as a result of nuclear translocation of transcription factors EB and E3 (27). To determine if this also occurred during infection with *L. mexicana*, the proteomics data set was overlaid with the Gene Ontology (GO) terms, “lysosome,” “lysosome lumen,” or “lysosome membrane” (GO:0005764, GO:0043202, or GO:0005765, respectively). Unlike what has been reported for *C. burnetii* infection, there was no increased abundance of lysosomal proteins in cells infected with *L. mexicana* (Fig. 5B, pink circles). Specific examination of cathepsin abundance also revealed that cathepsins were not significantly altered during *L. mexicana* infection, consistent with our earlier findings (Fig. 5B, labels). This suggests that while *L. mexicana* resides in a phagolysosomal niche, there is likely no associated increase in lysosomal biogenesis.

The proteomics analysis was extended to the conditioned media of *L. mexicana*-infected cells to determine if lysosomal content was secreted to the extracellular space during infection, as observed for *C. burnetii*. 1,642 proteins were identified and quantified in the conditioned media fraction, 64 of which were significantly enriched in abundance during *L. mexicana* infection (as determined using a Student’s *t*-test with *P* < 0.05 and log_2_ fold change > 1). As before, PCA confirmed that biological replicates from each group clustered together (Fig. 5C). There was no significant enrichment of lysosomal proteins in the media following *L. mexicana* infection (Fig. 5D, pink circles). Further to this, cathepsins were broadly unchanged in abundance in the media from *L. mexicana-*infected cells when compared to media harvested from uninfected cells (Fig. 5D, labels). Collectively, this demonstrates that secretion of lysosomal content is not observed upon infection with all intralysosomal pathogens.

Finally, cathepsin secretion following co-infection of cells with both *C. burnetii* and *L. mexicana* was examined. Immunoblotting revealed that, consistent with previous data, *C. burnetii* infection induced a significant increase in the secretion of cathepsins B and D relative to uninfected cells (Fig. 5E, F, and G). This increase was not observed during infection of cells with *L. mexicana* alone, aligning with the mass spectrometry results. Interestingly, when cells were co-infected with both pathogens at equal MOI, *C. burnetii* appeared to dominate with respect to cathepsin trafficking (Fig. 5E, F, and G). In cell lysates, cathepsin B was removed, and immature cathepsin D (pCTSD) accumulated, while in conditioned media, both cathepsins were increased in abundance. Overall, the findings presented here imply that modulation of cathepsins is not shared between all intralysosomal pathogens. Instead, it is a specific virulence strategy induced by *C. burnetii*, while *L. mexicana* amastigotes are able to tolerate exposure to the full repertoire of host lysosomal proteases.

## DISCUSSION

The lysosome represents a key innate immune strategy for the removal of invading pathogens. Yet, a unique subset of intracellular pathogens can avoid degradation by these mechanisms and replicate in phagolysosomal compartments. We recently reported that the intralysosomal bacterium *Coxiella burnetii*, the causative agent of Q fever, strategically modifies the protease repertoire of the lysosome by removing the key enzyme cathepsin B to promote bacterial success (20). In the present study, we examined whether this extended to *Leishmania*, a protozoan parasite that also resides within an intralysosomal niche.

We examined the abundance and activity of cathepsin proteases during *C. burnetii* or *L. mexicana* infection using immunoblotting and ABP analysis. Loss of cathepsin B during *C. burnetii* infection was consistently observed; however, this was not the case for cells infected with *L. mexicana* (Fig. 1). In fact, the protease cohort of *L. mexicana-*infected cells appeared broadly unchanged from uninfected cells. This suggests that cathepsin B removal is a specific virulence strategy employed by *C. burnetii* to promote intracellular success. Although the vacuoles of *C. burnetii* and *L. mexicana* are alike in being fusogenic, acidic compartments, there is existing literature to suggest that these two pathogens exert differing effects on the host cell (28). The present study aligns with these observations, adding that this extends to modulation of the host lysosomal protease cohort. Furthermore, this examination of protease abundance and activity in *L. mexicana* infection is also consistent with previous reports using *L. amazonensis*, which showed that lysosomal hydrolase activity remains constant or is marginally elevated during infection of bone-marrow-derived macrophages (29).

A question that remains to be addressed is whether the lysosomal environment is altered during *L. mexicana* infection. Early studies showed that surface lipophosphoglycan (LPG) of promastigote stages of *L. donovani* and *L. major* can delay phagosome maturation after entry into macrophages, allowing time for parasites to differentiate to the hydrolase-resistant amastigote form (30). Phagosome maturation was delayed, in part, by exclusion of v-ATPase, allowing *Leishmania* promastigotes to establish infection in a non-acidified compartment (31). However, other species of *Leishmania* (e.g., *L. mexicana*) are not dependent on LPG for promastigote infection and intracellular development, indicating species-specific mechanisms for avoiding or preventing lysosomal killing (32). Moreover, in contrast to promastigotes, *Leishmania* amastigotes lack LPG (33), and there is no evidence that these stages induce delayed phagolysosome maturation in any species (10). Based on the findings of this study, we propose that neither promastigotes nor amastigotes of *Leishmania* are dependent on the down-regulation of host cathepsin proteases for intracellular survival.

Here, both co- and super-infection models were established to determine if the presence of both pathogens inside a cell might alter cathepsin B abundance or pathogen proliferation. In these models, co-infected cells appeared to display the same cathepsin phenotypes as cells infected with *C. burnetii* alone, suggesting that *C. burnetii* orchestrates changes to cathepsins that *L. mexicana* can withstand without any apparent fitness cost. Importantly, co-infection with *L. mexicana* did not rescue the loss of cathepsin B observed during *C. burnetii* infection. It should be acknowledged that the ability of these pathogens to replicate inside a THP-1 cell *in vitro* varies significantly. In existing literature, *C. burnetii* shows 500- to 1,000-fold replication across 5 days of infection (34, 35), while THP-1 cells only support a much smaller number (~20-fold replication) of *L. mexicana* parasites (36).

We observed a marginal increase in *C. burnetii icmL*::Tn replication inside pre-existing *L. mexicana* vacuoles (Fig. 4B, C, and E). This is attributed to a small proportion of *L. mexicana*-infected cells supporting replication of the Dot/Icm-deficient *C. burnetii* (Fig. 4E). Given that *L. mexicana* does not induce loss of cathepsin B, it is interesting to consider whether this phenotype would be amplified in cathepsin B-deficient cells, leading to full complementation of the replication defect observed with the *icmL*::Tn mutant. In our prior study, overexpression of cathepsin B induced only a partial defect in replication of *C. burnetii*. Although unlikely to be sufficient to completely rescue the severity of the defect observed for *icmL*::Tn mutants, future studies may consider investigating replication kinetics in cathepsin B-deficient cells.

Although *C. burnetii* and *L. mexicana* show similarities in their intracellular niches, it is interesting to consider the key differences between these two pathogens. Specifically, *Leishmania* amastigotes switch to a slow growth state with greatly reduced rates of metabolic activity compared to extracellular promastigotes, which is associated with increased resistance to a wide variety of cellular and oxidative stresses (reviewed in references 37, 38). Interestingly, both pathogens are auxotrophic for many amino acids, and it will be of interest to determine whether alterations in proteolytic capacity within the phagolysosome are associated with changes in the availability of free amino acids/peptides. The lysosome-localized host mechanistic target of rapamycin complex 1 (mTORC1) is a key regulator of cellular nutrient sensing but also has a role in lysosomal homeostasis by controlling the activity of TFEB, the major regulator of lysosomal biogenesis and autophagy (39, 40). Under nutrient-rich conditions, mTORC1 phosphorylates TFEB to keep it retained in the cytoplasm, maintaining homeostasis by preventing excess lysosomal biogenesis and autophagy. However, the *Leishmania* protease GP63 cleaves mTOR, preventing proper host protein translation and effectively reducing the host immune response (41). Theoretically, mTOR cleavage by *Leishmania* should result in TFEB dephosphorylation and nuclear translocation, culminating in increased lysosomal biogenesis and autophagy. Although we did not directly examine TFEB activity in this study, the proteomics analysis performed in Fig. 5 did not show increased abundance of lysosomal proteins during *L. mexicana* infection. It is therefore interesting to consider whether other regulatory pathways such as calcium/calcineurin signaling, JNK signaling, or the AMP-activated protein kinase pathway contribute to *Leishmania* modulation of the host cell.

Collectively, alterations to lysosomal biology that are induced by *C. burnetii* are not observed during infection with *L. mexicana*. A potential rationale for this is that the unique metabolic state of *Leishmania* allows it to withstand the harsh phagolysosomal environment, while *C. burnetii* instead resorts to altering the environment itself through modulation of key lysosomal enzymes. Cumulatively, the findings presented here provide deeper insight into how these two “intralysosomal” pathogens differ in their mechanisms of withstanding this hostile environment.

## MATERIALS AND METHODS

### Propagation of *C. burnetii* and *L. mexicana*

Axenic cultures of *C. burnetii* Nine Mile Phase II RSA439 were generated in acidified cysteine citrate media 2 (42, 43) for 6–7 days at 37°C with 5% CO_2_ and 2.5% O_2_. Where required for cultivation of *C. burnetii icmL*::Tn, cultures were supplemented with kanamycin at 350 μg/mL.

Promastigote cultures of *L. mexicana* were grown in Roswell Park Memorial Institute (RPMI) media supplemented with 10% heat-inactivated fetal calf serum (FCS). Promastigotes were maintained at 27°C and passaged every 3–4 days.

*L. mexicana* amastigotes were generated by transferring stationary-phase promastigotes to RPMI-HCl (pH 5.5) with 20% FCS and incubating at 33°C, 5% CO_2_ for 4 days prior to infection.

### Mammalian cell culture

THP-1 human monocytic cells were maintained in RPMI media with 10% FCS at 37°C with 5% CO_2_. Cells were differentiated to macrophage-like cells with phorbol 12-myristate 13-acetate (PMA) at a concentration of 10^−8^ M for 72 h prior to infection.

### Immunoblotting

At the desired time points post-infection, cells were washed with PBS and harvested in 2× Laemmli buffer for SDS-PAGE. Samples were boiled at 95°C for 10 min before being electrophoresed on an Any kD Mini-PROTEAN TGX Stain-Free protein gel (Bio-Rad) at 165 V for 45 min. Gels were transferred to PVDF membranes and blocked for 1 h in 5% skim milk in Tris-buffered saline + 0.1% Tween-20 (TBST). Primary and secondary antibodies (see below) were diluted in 5% bovine serum albumin (BSA) in TBST. Blots were imaged on an AI680 Imager (GE Healthcare) or a ChemiDoc MP Imager (Bio-Rad) using the Western ECL Clarity substrate (Bio-Rad).

Antibodies used in this study are as follows: anti-Cathepsin B (Cell Signaling Technology, #31718, 1:2,000), anti-Cathepsin C (Santa Cruz Biotechnology, #sc-74590, 1:2,000, anti-Cathepsin D (Abcam, #72915, 1:5,000), Actin (Sigma, #A1978, 1:10,000).

### ABP analysis

At the desired time point, cells were harvested by replacing media with PBS and using a cell scraper to collect. Cells were pelleted by centrifugation at 7,000 × *g* for 5 min. Cells were lysed in a citrate buffer (50 mM citrate, pH 5.5, 0.5% CHAPS, 0.1% Triton-X 100, 4 mM DTT) and total protein was quantified using a BCA assay (Pierce). BMV109 (cathepsins B, S, X, L) was added to lysates (1 µM) and incubated for 45 min at 37°C. The reaction was stopped by addition of sample buffer and boiling for 5 min. Proteins were resolved via SDS-PAGE and in-gel fluorescence was detected by scanning on a Typhoon imager (GE Healthcare) using a Cy5 filter. When required, gels were then transferred to nitrocellulose membranes and immunoblotted as above.

### Infection of THP-1 cells with *L. mexicana* and/or *C. burnetii*

THP-1 cells were plated at a density of 5 × 10^5^ cells per well in a 24-well plate and differentiated with PMA as above.

For *C. burnetii* infections, stationary-phase (6 or 7 day) axenic cultures were pelleted and resuspended in RPMI + 10% FCS before being quantitated using qPCR and primers specific to *ompA*. Concentration of the bacterial culture was determined by comparison against a standard curve. *C. burnetii* was added to THP-1 cells at MOI 10 before culture plates were centrifuged for 30 min at 500 × *g*. Cells were then washed thrice with PBS and incubated at 37°C, 5% CO_2_ until the desired time point.

For *L. mexicana* infections, amastigote cultures were quantified visually using a Neubauer hemocytometer. Parasite suspensions were added to cells at an MOI of 5 or 10 before plates were centrifuged at 300 × *g* for 5 min to promote contact between amastigotes and THP-1 cells. Cells were left to incubate at 33°C with 5% CO_2_ for 4 h before being washed thrice with PBS and returned to 33°C, 5% CO_2_ for the desired time.

For co-infections, bacteria and parasites were individually quantified as above. THP-1 cells were either infected with both organisms simultaneously (co-infection) or infected with *L. mexicana* and left for 3 days at 33°C before being infected with *C. burnetii* (super-infection).

### Quantification of intracellular replication

Quantitative PCR was used to determine intracellular replication of *C. burnetii* and *L. mexicana* within THP-1 cells. Immediately following infection (D0) or at days 1, 3, and 5 post-infection, cells were osmotically lysed in dH_2_O and collected by centrifugation at 17,000 × *g* for 15 min. Genomic DNA was extracted using Quick DNA Miniprep Kit (Zymo Research) according to the manufacturer’s protocol. qPCR was performed using the SensiFast SYBR No-Rox kit (Bioline) and primers specific to either *ompA* (for *C. burnetii*) or *its1* (for *L. mexicana*). Serial dilutions of *C. burnetii* or *L. mexicana* were used to generate a standard curve for quantitation. qPCR was performed using a QuantStudio 7 Flex RT-qPCR (Thermo Fisher Scientific).

Oligonucleotides used for quantification are as follows: OmpA_F CAGAGCCGGGAGTCAAGCT, OmpA_R CTGAGTAGGAGATTTGAATCGC, Its1_F GGATCATTTTCCGATGATTACACC (26), Its1_R CTGCAAATGTTGTTTTTGAGTACA (26).

### Fluorescence microscopy

Differentiated THP-1 cells seeded on glass coverslips were infected with *L. mexicana,* expressing TurboRFP (44), at an MOI of 10 for 3 days, then super-infected with *C. burnetii* WT or *icmL*::Tn strains at an MOI of 10 for a further 5 days, as outlined above. Cells were then washed with PBS and fixed with 4% paraformaldehyde for 15 min, washed three times with PBS, then permeabilized with PBS + 1% BSA (Roche) and 0.2% Triton X-100 for 15 min. Cells were washed with PBS and blocked for 1 h in PBS + 0.1% Tween-20 and 5% normal goat serum (blocking buffer). All antibodies used for immunofluorescence were diluted in blocking buffer. Rabbit anti-*C-burnetii* (in-house, 1:10,000) and mouse anti-LAMP-1 (Developmental Studies Hybridoma Bank, 1:100) were added to cells for 2 h at room temperature, then cells were washed three times in TBST, and Goat anti-mouse488 (Thermo Fisher, 1:1,000) and Goat anti-rabbit647 (Thermo Fisher, 1:1,000) were added for 1 h at room temperature. Coverslips were stained with 4ʹ,6-diamidino-2-phenylindole (DAPI, Life Technologies) before five additional washes in TBST and mounting with ProLong Diamond (Life Technologies). Samples were imaged on a Leica Stellaris 5 inverted confocal microscope with a 63× oil-immersion objective. Images were acquired as a series of five sections in the *z*-axis and are presented as maximum projections of z-stacks. Processing of confocal imaging was performed with FIJI (45).

### Mass spectrometry on *L. mexicana*-infected cell lysates and culture media

#### Sample collection

THP-1 cells were plated in 10 cm dishes at a density of 1.1 × 10^7^ cells per dish and infected with *L. mexicana* at an MOI of 25, as described above. Four biological replicates were prepared for each condition (uninfected and infected, respectively). After 48 h of infection, cells were washed once with PBS and incubated in fresh RPMI containing no FCS (serum-free) for a further 18 h. After this time, supernatants were collected and centrifuged at 3,000 × *g* for 10 min to pellet any cell debris. The supernatants were then concentrated through Amicon Ultra Centrifugal Filters (Merck) with a molecular weight cut-off of 10 kDa, before being combined with a lysis buffer containing 10% sodium dodecyl sulfate and 100 mM Tris-HCl, pH 8.0. Simultaneously, cell fractions were washed thrice with PBS before being lysed in the same lysis buffer. All samples were then incubated at 95°C with shaking at 2,000 rpm to shear DNA before total protein was quantitated using Nanodrop. Mass spectrometry sample preparation was performed using 100 μg of protein as input.

#### Sample preparation

Dithiothreitol (DTT) was added to a final concentration of 10 mM, and samples were incubated at 95°C for 10 min before alkylation was performed using 40 mM iodoacetamide at room temperature in the dark. After 30 min, the reaction was quenched with the addition of another 10 mM DTT, then samples were acidified with 12% phosphoric acid. Samples were then combined with a binding/wash buffer (100 mM Tris (pH 8.0), 90% (vol/vol) methanol) before being loaded onto S-trap micro columns (ProtiFi, USA). Columns were washed four times with the same binding/wash buffer before samples were digested with SoluTrypsin (Sigma) using a 3 µg trypsin per sample (approximately 1:33 trypsin:input), diluted in 50 mM Tris-HCl, pH 8. Digestion was performed at 37°C in a humidified container for 18 h. The following day, samples were eluted by centrifugation using 50 mM Tris-HCl, pH 8, followed by 0.2% formic acid, then with 50% acetonitrile and 0.2% formic acid at 4,000 × *g* for 1 min each. The combined eluate was collected and vacuum-dried before being resuspended in mass spectrometry loading buffer (“Buffer A*,” 0.1% trifluoroacetic acid, 2% acetonitrile) and subjected to desalting using C_18_ stage tips (46, 47).

### Liquid chromatography mass spectrometry

C_18_-enriched proteome samples were re-suspended in Buffer A* and separated using a two-column chromatography setup composed of a PepMap100 C_18_ 20 mm by 75 mm trap (Thermo Fisher Scientific) and a PepMap C_18_ 500 mm by 75 mm analytical column (Thermo Fisher Scientific) using a Dionex Ultimate 3000 UPLC (Thermo Fisher Scientific). Samples were concentrated onto the trap column at 5 µL/min for 6 min with Buffer A (0.1% formic acid, 2% DMSO) and then infused into an Orbitrap Fusion Lumos (Thermo Fisher Scientific) at 300 nL/min via the analytical columns. Peptides were separated by altering the buffer composition from 3% Buffer B (0.1% formic acid, 77.9% acetonitrile, 2% DMSO) to 23% B over 70 min, then from 23% B to 40% B over 4 min, and then from 40% B to 80% B over 3 min. The composition was held at 80% B for 2 min before being returned to 3% B for 10 min. The Orbitrap Fusion Lumos Mass Spectrometer was operated in a data-dependent mode, automatically switching between the acquisition of a single Orbitrap MS scan (300–2,000 m/z, maximal injection time of 50 ms, an Automated Gain Control (AGC) set to a maximum of 400k, and a resolution of 60k) and 3 s of Orbitrap MS/MS HCD scans of precursors (NCE 35%, a maximal injection time of 80 ms, an AGC of 125,000 and a resolution of 30k).

### Data analysis

Raw spectral files were searched using FragPipe (48, 49) (v.22.0) against *Homo sapiens* (UniProt accession: UP000005640) and *Leishmania mexicana* (UniProt accession: UP000007259) reference proteomes. FragPipe parameters were left on default unless otherwise specified. Label-free quantification (LFQ) was performed allowing for cysteine carbamidomethylation as a fixed modification (+57.0215 Da), N-terminal acetylation (+42.0106 Da), and oxidation of methionine (+15.9949 Da) as variable modifications. Protease specificity was “strict trypsin”’ with cleavage at K or R residues, and two missed cleavages (maximum) were allowed. Philosopher was used to calculate protein and peptide FDRs. Resulting data were further analyzed using the Perseus (50) (v.1.6.0.7) software. Data were subject to log_2_ transformation and then filtered to only retain proteins with valid values identified in at least three out of four biological replicates in at least one group (i.e., uninfected/infected). Missing values were imputed based on a downshifted normal distribution (width = 0.3σ, downshift = −1.8σ). Statistical comparison between groups was performed using a Student’s *t*-test and multiple hypothesis correction based on permutation-based FDR. Data visualization was performed using the ggplot2 package in R (v.4.2.1).

## Data Availability

Mass spectrometry raw data files have been uploaded to the ProteomeXchange consortium via the PRIDE (51) repository with the data set identifiers PXD063745.

## SUPPLEMENTAL MATERIAL

Data S1 iai.00270-25-s0001.xlsx Mass spectrometry data.

ASM does not own the copyrights to Supplemental Material that may be linked to, or accessed through, an article. The authors have granted ASM a non-exclusive, world-wide license to publish the Supplemental Material files. Please contact the corresponding author directly for reuse.
